# Supplementary material for: HIV-1-mediated insertional activation of STAT5B and BACH2 trigger viral reservoir in T regulatory cells
Source: Nat Commun. 2017 Sep 8;8:498. doi: 10.1038/s41467-017-00609-1 (PMC5591266; doi:10.1038/s41467-017-00609-1)
Supplement: Supplementary file 1 — Supplementary Information [file 41467_2017_609_MOESM1_ESM.pdf]

File name: Supplementary Information
Description: Supplementary Figures, Supplementary Tables, Supplementary Methods and Supplementary References

File name: Peer Review File

Supplementary Figure 1

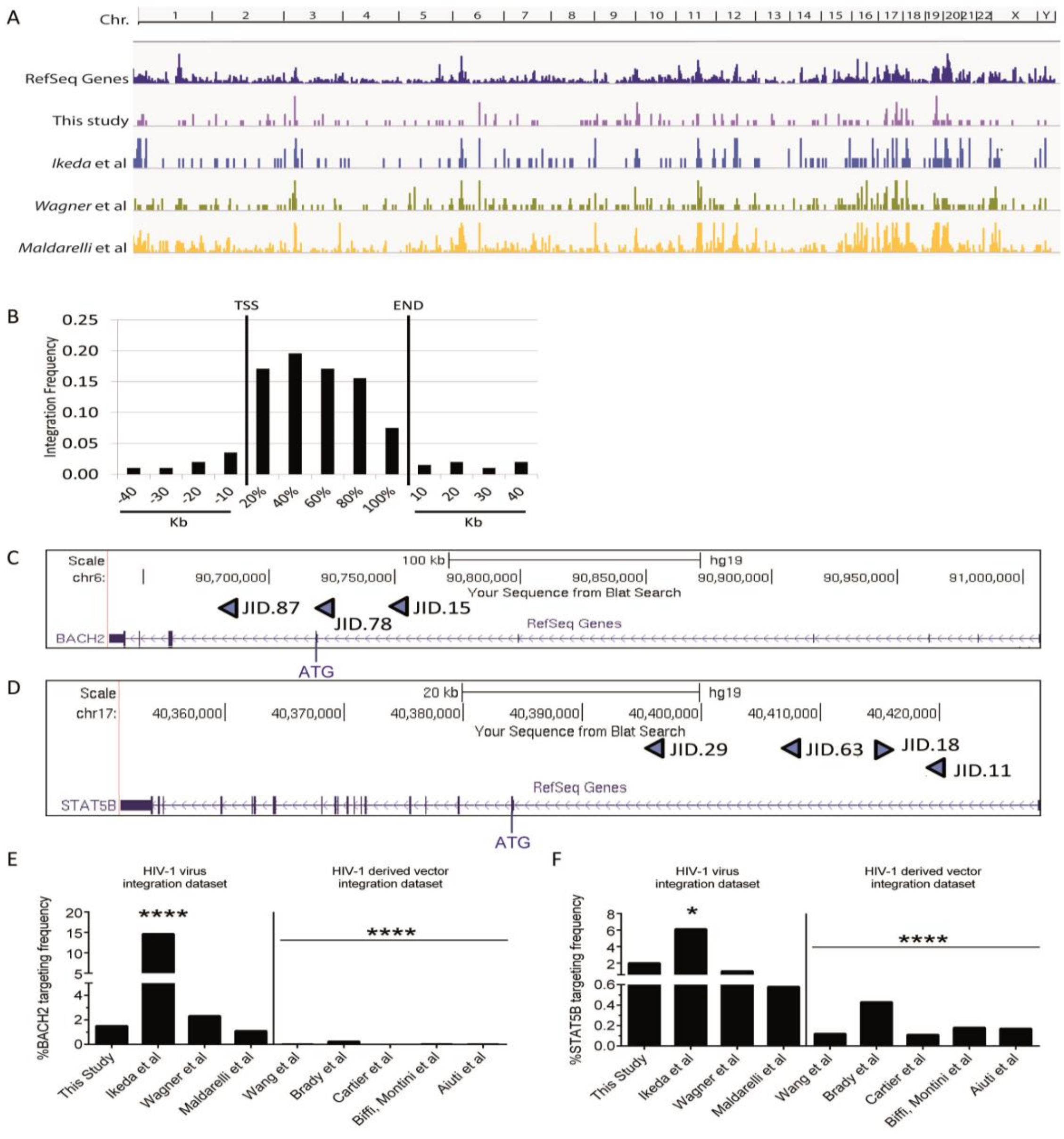

Supplementary Figure 1: HIV-1 integrations targeting *BACH2* and *STAT5B*. A) Frequency distribution along chromosomes (top track) of RefSeq genes (second track below), and for the indicated HIV-1 virus integration dataset; B) Frequency distribution of the HIV-1 virus integrations retrieved in this study with respect to the targeted gene transcription unit: for each gene its length was divided in five intervals and for each intervals the frequency was calculated by numbering the fraction of integrations mapping in each of those. Integrations upstream and downstream of genes are shown in 10 Kb intervals. C, D) HIV-1 integration sites targeting *BACH2* (C) and *STAT5B* (D) found in PBMCs of a cohort of 54 patients under cART (see Supplementary Table 1 for details). For both genes, genomic coordinates and scale are indicated. Blue boxes and bars indicate exons, blue arrows indicate the orientation of gene transcription, blue triangles above each gene indicate position and orientation of the integration site. Patient ID for each integration site is indicated near the arrow. E, F) Targeting frequency of *BACH2* (E) and *STAT5B* (F) in different HIV-1 virus or vector derived integration dataset. In both histograms, the work from which each integration dataset was retrieved is indicated on the x-axis. P values were defined by Fisher exact test; see Supplementary Table 4 for details and number.

Supplementary Figure 2

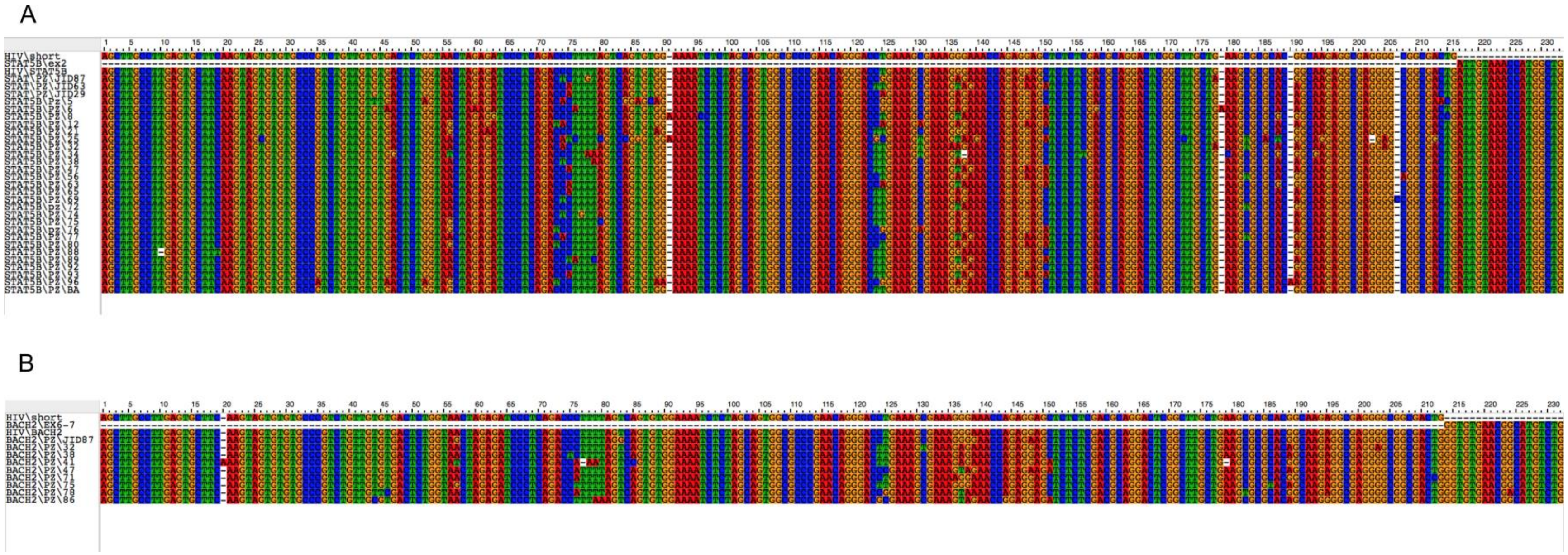

Supplementary Figure 2: Sequences of the chimeric HIV/*STAT5B* and HIV/*BACH2* transcript from infected patients. Multiple sequence alignments of the chimeric HIV/*STAT5B* A) and HIV/*BACH2* B) transcripts found in different patients. In each panel, the first row represents the HIV-1 sequence used as reference standard (HXB2 from the HIV database www.hiv.lanl.gov), the second row represents the sequence of the first protein coding exon of the targeted gene (Ex2 for *STAT5B* or Ex6 for *BACH2*) and the third row represents the theoretical chimeric sequence that should be formed from the fusion of the HIV-1 SD signal and nearby coding exon of the targeted gene. All the remaining rows represent in A) HIV/*STAT5B* chimeric sequences and in B) HIV/*BACH2* chimeric sequences identified in HIV-1 infected patients, as indicated.

Supplementary Figure 3

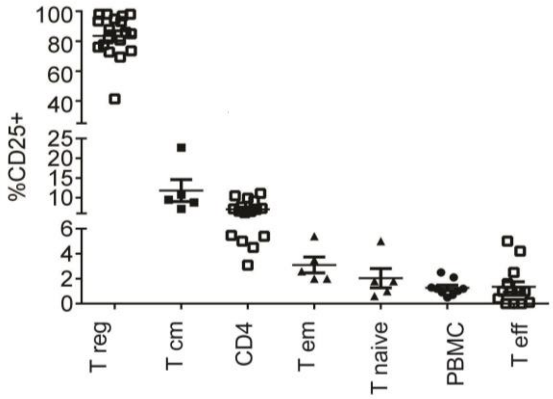

Supplementary Figure 3: Relative percentage of the amount of CD25+ cells within the different T cell subsets, as indicated.

Supplementary Figure 4

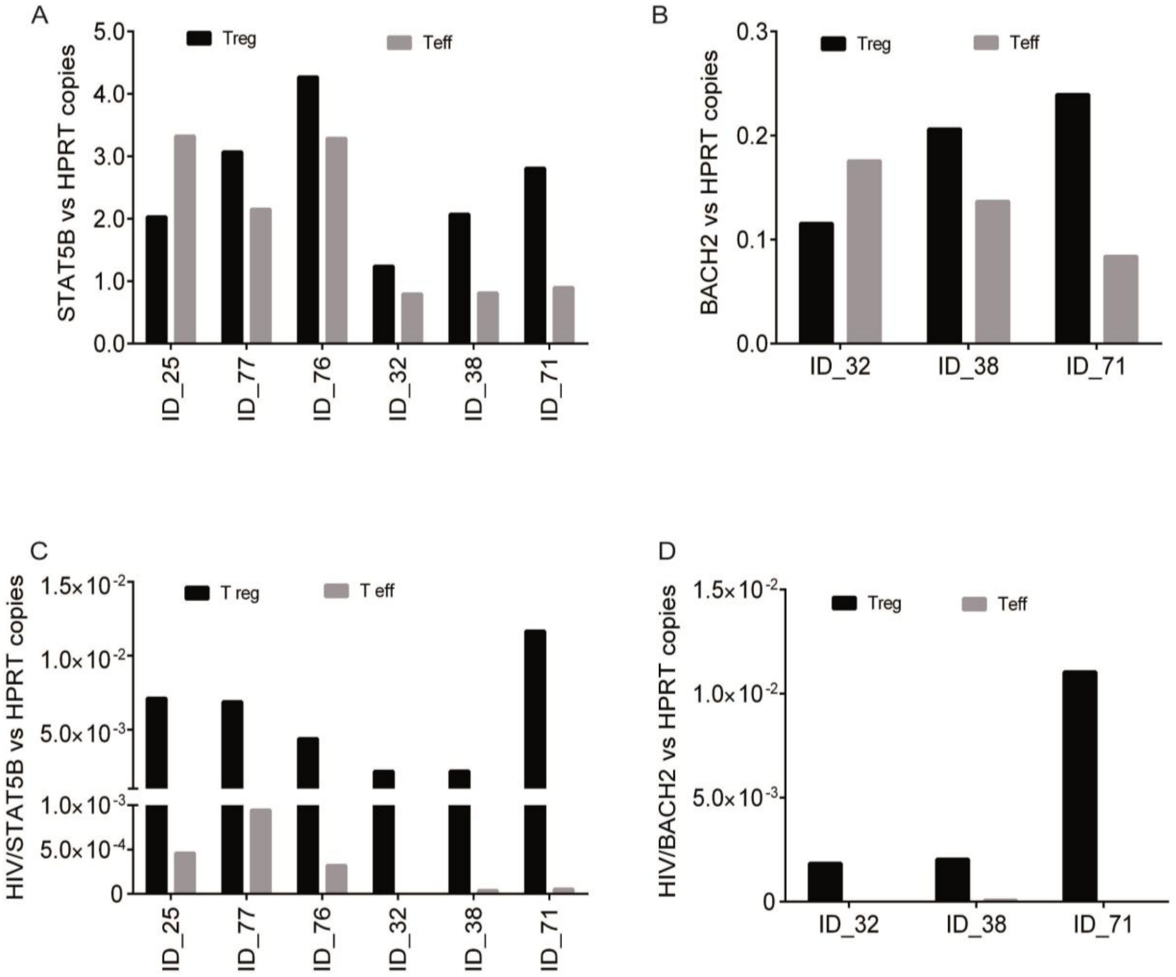

Supplementary Figure 4: Quantitative assessment of the chimeric HIV/*STAT5B* and HIV/*BACH2* chimeric transcript in the Treg and T eff compartment by ddPCR. A, B) Histograms indicate the relative levels of the copies of *STAT5B* (A) and of BACH2 (B) versus *HPRT* copies measured by ddPCR in Treg (black bar) and T eff (grey bar) cells obtained from the blood of each single HIV-infected patients analyzed. C, D) Histograms indicate the relative levels of the copies of HIV/*STAT5B* (C) and of HIV/*BACH2* (D) versus *HPRT* copies measured by ddPCR in Treg (black bar) and Teff (grey bar) cells obtained from the blood of each single HIV-infected patients analyzed. Of note, since only a small proportion of the entire Treg population should harbor the HIV integration within the host gene, the level of expression of the HIV/*STAT5B* and of HIV/*BACH2* chimeric transcript is lower compared to the level of expression of *STAT5* and of *BACH2*.

Supplementary Figure 5

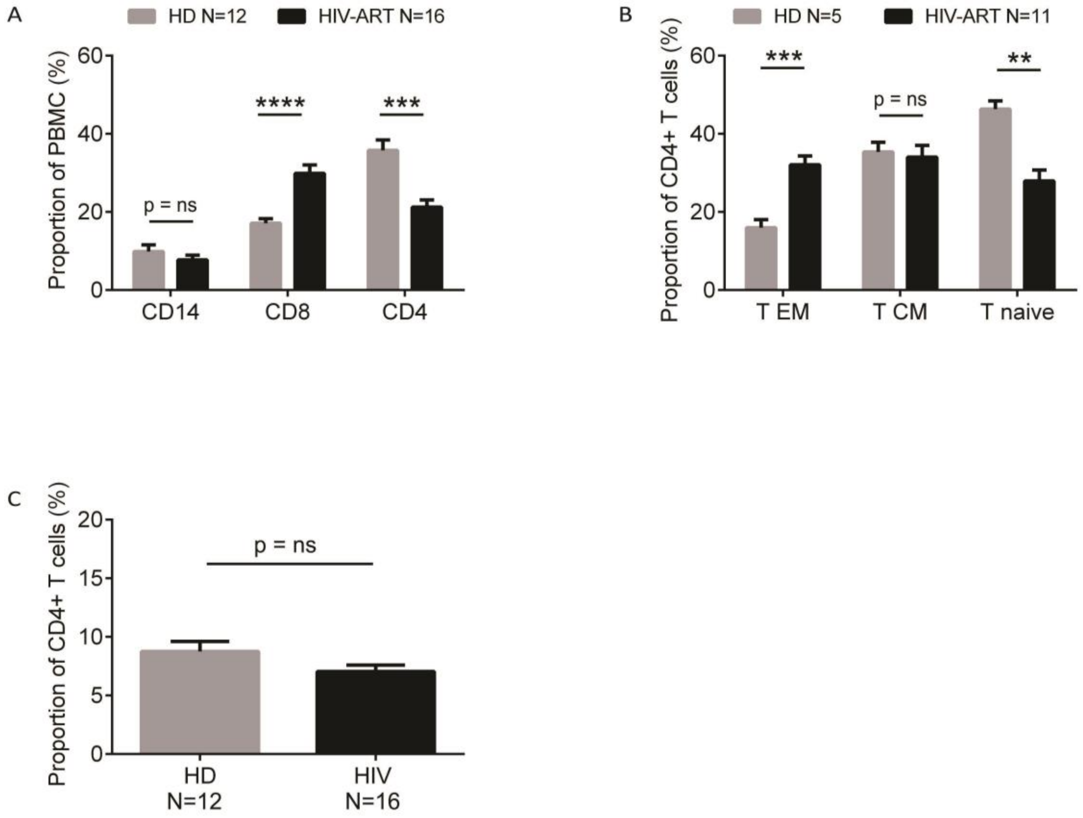

Supplementary Figure 5: Proportions of different hematopoietic cell subsets in healthy donors (HIV negative subject) and HIV patients under ART expressing at least one of the chimeric transcripts in total PBMC. A) Proportions of CD14+, CD8+ and CD4+ cells in healthy donors (HD, N=12) and in ART-treated HIV positive individuals (HIV-ART, N=16) in which we detect the presence of the chimeric mRNAs from total PBMC. Data are represented as relative proportion of total PBMC; B) Proportions of Tem, Tcm and Tnaive cells within CD4+ cells and detected in healthy donors (HD, N=5) and in ART-treated HIV positive individuals (HIV-ART, N=11) in which we detect the presence of the chimeric mRNAs from total PBMC. C) Relative proportion within total CD4+ T cells of Treg cells in healthy donors (HD, N=12) and ART-treated individuals (HIV-ART, N=16) are shown. Data are represented as Mean ± SEM. Significance was tested using unpaired t test on Log-ODD transform % values (**, ***, **** reflect $p<0.01$, $p<0.001$ and $p<0.0001$ respectively).

Supplementary Figure 6

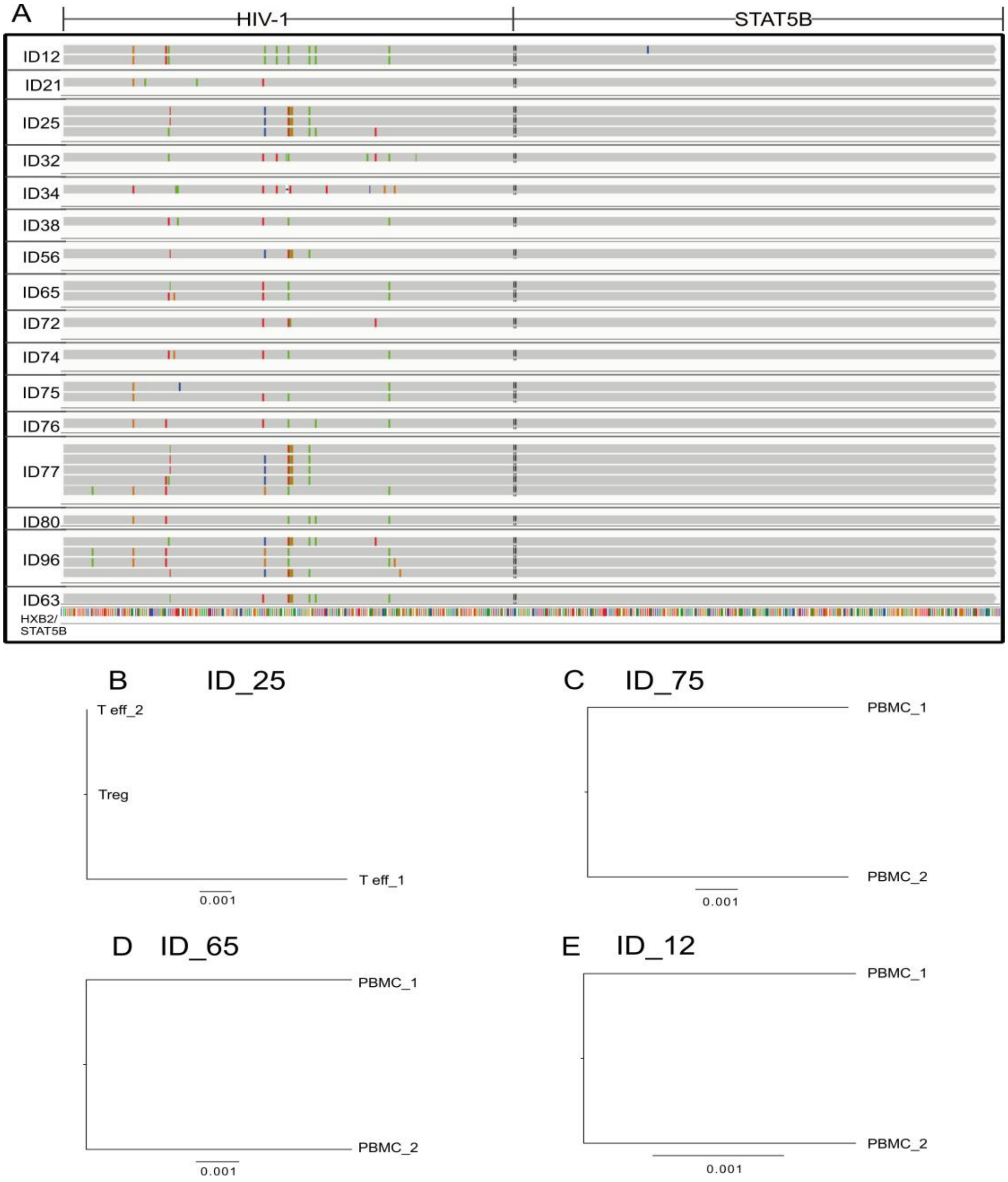

Supplementary Figure 6: Phylogenies of amplified chimeric HIV/*STAT5B* bands. A) Alignment of of the HIV-1/*STAT5B* haplotypes identified in each patient. The reference HIV-1 sequence (HXB2 from the HIV database, www.hiv.lanl.gov) spanning from the U5 to the major SD signal (212 bp) and joined to the *STAT5B* portion (exons 2, 3 and 4) is reported at the bottom of the graph. For each patient (ID indicated on the left) one or more haplotypes are shown; colored bars identified nucleotide variants respect to the reference sequence: green bars stand for Adenine, orange for Guanine, blue for Cytosine and red for Thymidine; Black bars divides the HIV-1 portion of the chimeric transcript from the *STAT5B* one. B-E) Maximum-likelihood trees constructed aligning the haplotypes identified analyzing the HIV-1 sequence from the U5 to the major SD signal among different hematopoietic cell subsets for ID_25, ID_75, ID_56 and ID_65. The hematopoietic cell subset from which that specific haplotype was identified is indicated at each branch tip of the tree. The number indicates if different haplotypes have been identified within the same hematopoietic cell subsets. Branch length varies according to the number of substitutions as a proportion of the length of the alignment (Distance value). Scale bar is reported below each tree.

Supplementary Figure 7

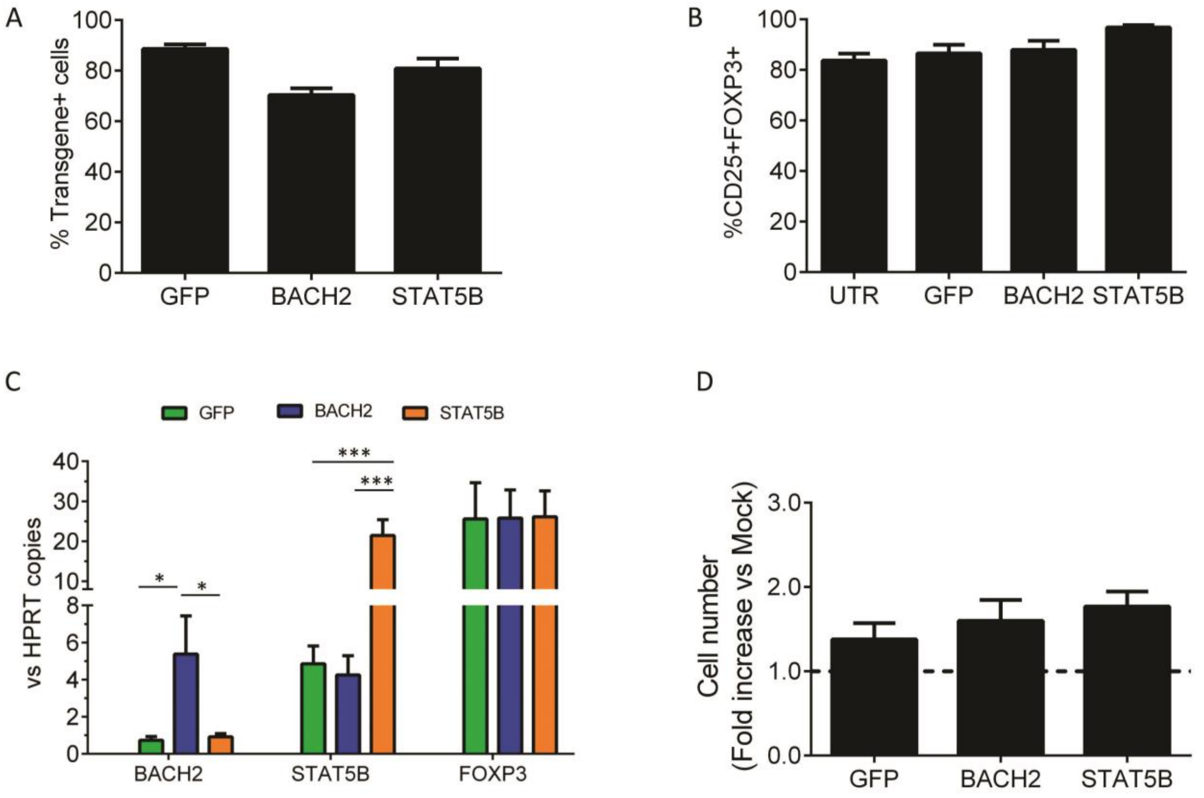

Supplementary Figure 7: Effects of forced expression of *STAT5B* and *BACH2* in freshly isolated Treg cells. A) Relative percentage of GFP+ or Orange+ cells (for bidirectional SIN LVs encoding for *BACH2* or *STAT5B*) of Treg transduced with the indicated vector 5 days after transduction, N=4; B) Relative percentage of CD25+FOXP3+ cells measured in Treg cells after 10 days of culture in presence of Treg expander beads and IL-2 (100 U/ml), N=4; C) Gene expression levels of *BACH2*, *STAT5B* and *FOXP3* measured by dd PCR in Treg transduced with the indicated vector after ten days of expansion in culture. For each sample, the relative copies of *BACH2*, *STAT5B* and *FOXP3* are normalized to the level of the housekeeping gene *HPRT, N=7*; D) Fold increase in the amount of Treg cells transduced with the indicated vector after ten days of culture. Data are represented as Fold increase versus untrasduced control, N=4. Data are represented as Mean ± SEM. Significance was tested using One way Anova with Bonferroni post-test (*, ***, reflect $p<0.05$ and $p<0.001$ respectively).

Supplementary Figure 8

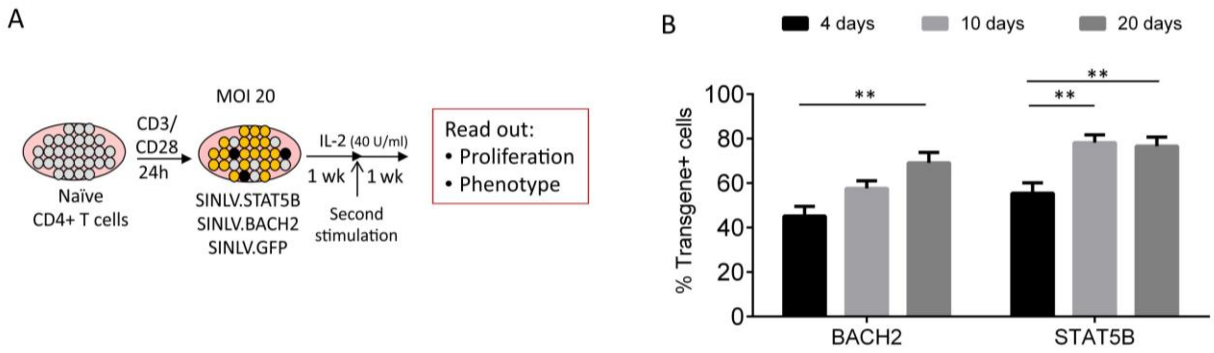

Supplementary Figure 8: Effects of enforced expression of *STAT5B* and *BACH2* in human naïve CD4+ T cells. A) Scheme of the experimental strategy. CD4+ naïve T cells from PBMC or cord blood mononuclear cells of healthy donors were activated by anti-CD3/CD28 beads for 24 hours, transduced with the indicated LVs and cultured in presence of low doses of IL-2 (40 U/ml); B) Percentage of transgene+ cells (Orange, for bidirectional LVs encoding for *BACH2* or *STAT5B*, GFP as marker for cells transduced with a control vector) measured over time in naïve CD4+ T cells transduced with the indicated vector, N=6. Data are represented as Mean ± SEM. Significance was determined by one way ANOVA with Bonferroni correction (*p<0.05; (**p<0.01)

Supplementary Figure 9

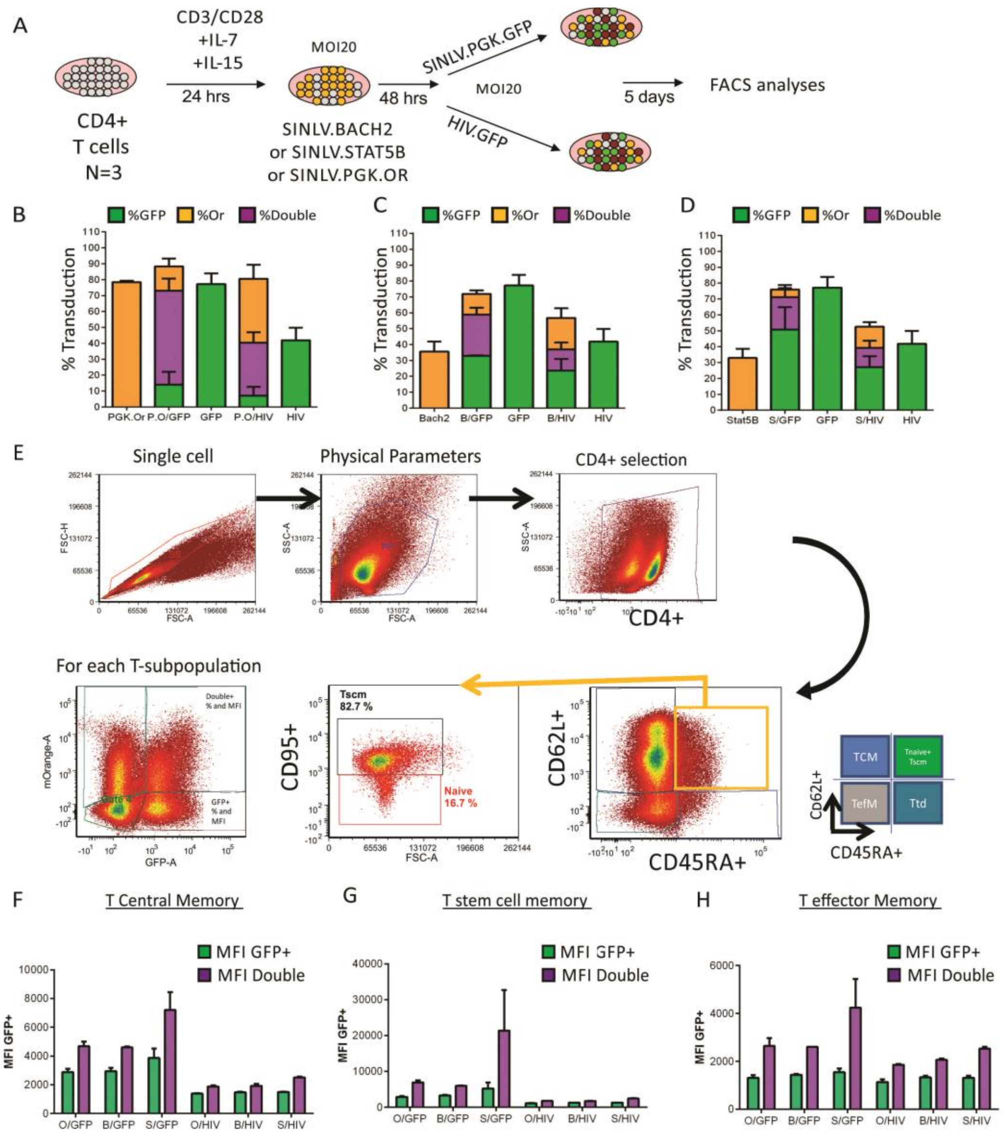

Supplementary Figure 9: Forced expression of *BACH2* and *STAT5B* do not affect HIV LTR activity. To address whether *STAT5B* and *BACH2* could silence HIV-1 genome expression, CD4+ T cells from healthy donors were transduced with the LVs expressing these transcription factors and then transduced with a replication defective tat-driven GFP-expressing HIV-1 vector containing all viral sequences, as transcriptional reporter (Browne, E. P. & Littman, D. R, Journal of Virology 2008). The GFP expression in the different T cell subsets of the in vitro culture was measured as readout of the transcriptional activity of the HIV-1 genome. A) Experimental strategy. CD4+ T cells were isolated from PBMC from healthy donors, activated for 24 hours with anti-CD3/CD28 expanding beads and in presence of recombinant human IL-7 and IL-15, and then infected at MOI 20 with the different bidirectional SIN LVs expressing *BACH2* or *STAT5B*. After 48 hours of transduction CD4+ T-cells were re-infected at MOI 20 with the HIV.GFP reporter vector or a PGK.GFP LV vector as control. Five days after the last transduction, cells were analysed by FACS. B, C, D) Column graph indicating the % of GFP+ (green), Orange (Or)+ (orange, for SINLV encoding for *BACH2* and *STAT5B*) or double positive cells (purple) for the different conditions. Cells were transduced with PGK (PGK.GFP (P.O/GFP) or HIV.GFP (P.O/HIV)) (B), SIN LV expressing *BACH2* (C) or *STAT5B* (D). . E) Dot plots demonstrating the sequential gating process used for the identification of T-naive, T-stem cell memory, T-central memory and T-effector memory cells. In each T-cell subsets, we compare the MFI of double positive GFP+/Or+ cells (Double) vs single GFP+ cells. F, G, H) MFI of double positive GFP+/Or+ cells (MFI Double) vs single GFP+ cells in the different T-cell subsets: (F) T-central memory and (G) T-stem cell memory and (H) T effector memory cells. Results are represented as Mean ± Standard Deviation, N=3

Supplementary Table 1. HIV-1 insertion sites obtained from total PBMC of infected patients

Patient ID	Treatment	Chr	Int Locus	ISS strand	Ref Seq Symbl	Gene Ori
JID.61	ART+IL2	1	19788236	+	CAPZB	-
JID.25	ART+IL2	1	23920129	+	MDS2	+
JID.46	ART+IL2	1	28398720	+	EYA3	-
JID.76	ART alone	1	31447123	-	PUM1	-
JID.76	ART alone	1	32510660	-	KHDRBS1	+
JID.11	ART+IL2	1	36010314	+	KIAA0319L	-
JID.11	ART+IL2	1	36701149	-	THRAP3	+
JID.72	ART alone	1	45925278	-	TESK2	-
JID.64	ART+IL2	1	155419317	-	ASH1L	-
JID.82	ART alone	1	167274057	+	POU2F1	+
JID.74	ART alone	1	176196223	+	RFWD2	-
JID.76	ART alone	1	203471022	+	OPTC	+
JID.7	ART+IL2	1	203471562	+	OPTC	+
JID.61	ART+IL2	2	7270465	+	RNF144A	+
JID.70	ART alone	2	26271947	-	RAB10	+
JID.19	ART+IL2	2	27897191	-	SLC4A1AP	+
JID.72	ART alone	2	61002748	-	PAPOLG	+
JID.63	ART+IL2	2	112403685	-	ANAPC1	-
JID.18	ART+IL2	2	118578818	-	DDX18	+
JID.74	ART alone	2	174806340	+	SP3	-
JID.91	ART alone	2	198031168	+	ANKRD44	-
JID.76	ART alone	2	202072536	-	CASP10	+
JID.66	ART+IL2	2	204032322	-	NBEAL1	+
JID.74	ART alone	2	208439316	-	CREB1	+
JID.58	ART+IL2	2	222293004	+	EPHA4	-
JID.89	ART alone	3	17443680	+	TBC1D5	-
JID.89	ART alone	3	17443812	-	TBC1D5	-
JID.84	ART alone	3	31657291	-	STT3B	+
JID.35	ART+IL2	3	47633760	+	SMARCC1	-
JID.55	ART+IL2	3	47687189	-	SMARCC1	-
JID.50	ART+IL2	3	48004651	+	MAP4	-
JID.15	ART+IL2	3	48102285	+	MAP4	-
JID.58	ART+IL2	3	48735000	+	IP6K2	-
JID.13	ART+IL2	3	52293430	+	WDR82	-

Patient ID	Treatment	Chr	Int Locus	ISS strand	Ref Seq Symbl	Gene Ori
JID.52	ART+IL2	3	113503711	+	ATP6V1A	+
JID.72	ART alone	3	128827425	+	RAB43	-
JID.18	ART+IL2	3	141144151	-	ZBTB38	+
JID.82	ART alone	3	141270061	+	RASA2	+
JID.7	ART+IL2	3	184618840	+	VPS8	+
JID.52	ART+IL2	3	195783976	-	TFRC	-
JID.76	ART alone	4	26279042	-	RBPJ	+
JID.34	ART+IL2	4	87473102	+	PTPN13	+
JID.13	ART+IL2	4	151860702	+	LRBA	-
JID.8	ART+IL2	4	164675902	+	MARCH1	-
JID.15	ART+IL2	5	23164243	+	CDH12	-
JID.18	ART+IL2	5	60215974	+	ERCC8	-
JID.34	ART+IL2	5	102929510	+	NUDT12	-
JID.17	ART+IL2	5	130703314	+	CDC42SE2	+
JID.67	ART alone	5	141797790	+	SPRY4	-
JID.76	ART alone	5	150131522	+	DCTN4	-
JID.47	ART+IL2	5	172431528	-	ATP6V0E1	+
JID.18	ART+IL2	6	24831813	+	FAM65B	-
JID.11	ART+IL2	6	34782431	-	UHRF1BP1	+
JID.74	ART alone	6	90461016	+	MDN1	-
JID.87	ART alone	6	90686472	-	BACH2	-
JID.78	ART alone	6	90725083	-	BACH2	-
JID.15	ART+IL2	6	90755051	-	BACH2	-
JID.11	ART+IL2	6	109341494	-	SESN1	-
JID.88	ART alone	6	111199807	-	AMD1	+
JID.11	ART+IL2	6	139627034	+	TXLNB	-
JID.11	ART+IL2	6	144882767	+	UTRN	+
JID.11	ART+IL2	6	144883067	-	UTRN	+
JID.15	ART+IL2	7	23399338	+	IGF2BP3	-
JID.64	ART+IL2	7	44667710	-	OGDH	+
JID.64	ART+IL2	7	45049901	+	CCM2	+
JID.15	ART+IL2	7	93760610	+	BET1	-
JID.51	ART+IL2	7	100145770	-	AGFG2	+
JID.69	ART alone	7	114684387	+	MDFIC	+
JID.41	ART+IL2	8	97298412	+	PTDSS1	+
JID.66	ART+IL2	8	101952613	+	YWHAZ	-
JID.79	ART alone	8	112943776	+	CSMD3	-

Patient ID	Treatment	Chr	Int Locus	ISS strand	Ref Seq Symbl	Gene Ori
JID.76	ART alone	8	117757697	+	EIF3H	-
JID.73	ART alone	8	121762929	+	SNTB1	-
JID.17	ART+IL2	8	130727525	+	GSDMC	-
JID.32	ART+IL2	8	141678751	-	PTK2	-
JID.49	ART+IL2	9	291011	+	DOCK8	+
JID.15	ART+IL2	9	5990133	+	KIAA2026	-
JID.63	ART+IL2	9	6440715	+	UHRF2	+
JID.85	ART alone	9	23849013	+	ELAVL2	-
JID.6	ART+IL2	9	31340660	+	ACO1	+
JID.11	ART+IL2	9	37183841	-	ZCCHC7	+
JID.8	ART+IL2	9	72922219	-	SMC5	+
JID.74	ART alone	9	73247082	-	TRPM3	-
JID.71	ART alone	9	82604979	-	TLE4	+
JID.72	ART alone	9	98684946	+	C9orf102	+
JID.85	ART alone	9	98714982	-	C9orf102	+
JID.76	ART alone	9	106864638	-	SMC2	+
JID.15	ART+IL2	9	113793864	+	LPAR1	-
JID.82	ART alone	9	132700084	+	FNBP1	-
JID.84	ART alone	9	139707345	+	C9orf86	+
JID.90	ART alone	9	139828817	-	FBXW5	-
JID.63	ART+IL2	9	139908796	+	ABCA2	-
JID.74	ART alone	9	140516196	+	EHMT1	+
JID.66	ART+IL2	10	4517223	-	LOC100216001	-
JID.88	ART alone	10	5834789	+	GDI2	-
JID.66	ART+IL2	10	43103736	-	ZNF33B	-
JID.18	ART+IL2	10	43896229	+	HNRNPF	-
JID.61	ART+IL2	10	70501149	-	CCAR1	+
JID.74	ART alone	10	74877796	+	NUDT13	+
JID.13	ART+IL2	10	75190728	+	ZMYND17	-
JID.76	ART alone	10	88225540	-	WAPAL	-
JID.83	ART alone	10	103569264	-	MGEA5	-
JID.75	ART alone	11	14352980	-	RRAS2	-
JID.55	ART+IL2	11	47845278	+	NUP160	-
JID.80	ART alone	11	62318545	-	AHNAK	-
JID.11	ART+IL2	11	62348619	-	TUT1	-
JID.46	ART+IL2	11	63988683	-	FERMT3	+
JID.83	ART alone	11	64556480	+	MAP4K2	-

Patient ID	Treatment	Chr	Int Locus	ISS strand	Ref Seq Symbl	Gene Ori
JID.17	ART+IL2	11	116914800	+	SIK3	-
JID.52	ART+IL2	11	118313017	-	MLL	+
JID.13	ART+IL2	11	119259455	-	USP2	-
JID.25	ART+IL2	12	7065681	+	PTPN6	+
JID.67	ART alone	12	7114069	-	LPCAT3	-
JID.16	ART+IL2	12	11817174	-	ETV6	+
JID.13	ART+IL2	12	12079983	-	ETV6	+
JID.70	ART alone	12	41918350	+	PDZRN4	+
JID.58	ART+IL2	12	44471314	+	TMEM117	+
JID.51	ART+IL2	12	46827163	-	SLC38A2	-
JID.52	ART+IL2	12	56434960	+	RPS26	+
JID.18	ART+IL2	12	56578819	+	SMARCC2	-
JID.34	ART+IL2	12	95782062	-	MIR331	+
JID.73	ART alone	12	112435997	+	TMEM116	-
JID.47	ART+IL2	12	122967384	-	ZCCHC8	-
JID.73	ART alone	13	41585078	-	ELF1	-
JID.29	ART+IL2	13	42871543	-	AKAP11	+
JID.13	ART+IL2	13	49669849	+	FNDC3A	+
JID.50	ART+IL2	13	96417350	-	DNAJC3	+
JID.84	ART alone	13	100815279	-	PCCA	+
JID.47	ART+IL2	13	113854231	+	PCID2	-
JID.52	ART+IL2	14	60739390	-	PPM1A	+
JID.50	ART+IL2	14	66843246	-	C14orf53	+
JID.74	ART alone	14	75901661	+	JDP2	+
JID.82	ART alone	14	104103484	+	KLC1	+
JID.11	ART+IL2	14	105902696	+	MTA1	+
JID.18	ART+IL2	15	40682347	-	C15orf23	+
JID.73	ART alone	15	41972935	-	MGA	+
JID.6	ART+IL2	15	56930592	-	ZNF280D	-
JID.73	ART alone	15	64825214	+	ZNF609	+
JID.13	ART+IL2	15	75824468	-	PTPN9	-
JID.81	ART alone	15	81298171	-	MESDC1	+
JID.73	ART alone	16	256621	+	LUC7L	-
JID.64	ART+IL2	16	4551554	+	HMOX2	+
JID.83	ART alone	16	34657506	-	LOC283914	+
JID.18	ART+IL2	16	89514869	-	ANKRD11	-
JID.61	ART+IL2	17	1364694	+	MYO1C	-

Patient ID	Treatment	Chr	Int Locus	ISS strand	Ref Seq Symbl	Gene Ori
JID.86	ART alone	17	2534316	-	PAFAH1B1	+
JID.78	ART alone	17	4601985	+	PELP1	-
JID.67	ART alone	17	5457742	+	NLRP1	-
JID.77	ART alone	17	6881388	+	ALOX12	+
JID.68	ART alone	17	28741019	-	CPD	+
JID.13	ART+IL2	17	37649046	-	CRKRS	+
JID.34	ART+IL2	17	38015488	+	IKZF3	-
JID.29	ART+IL2	17	40396646	-	STAT5B	-
JID.63	ART+IL2	17	40408188	-	STAT5B	-
JID.18	ART+IL2	17	40414546	+	STAT5B	-
JID.11	ART+IL2	17	40420450	-	STAT5B	-
JID.67	ART alone	17	42322412	-	SLC4A1	-
JID.66	ART+IL2	17	48807265	-	LUC7L3	+
JID.34	ART+IL2	17	54667878	-	NOG	+
JID.15	ART+IL2	17	54937524	-	DGKE	+
JID.39	ART+IL2	17	54959805	+	MTVR2	-
JID.71	ART alone	17	72466715	+	CD300A	+
JID.55	ART+IL2	17	73656519	-	RECQL5	-
JID.66	ART+IL2	17	73864606	+	TRIM47	-
JID.85	ART alone	17	76058516	+	TNRC6C	+
JID.18	ART+IL2	17	78740615	-	RPTOR	+
JID.91	ART alone	17	79553209	+	NPLOC4	-
JID.55	ART+IL2	18	12826921	+	PTPN2	-
JID.74	ART alone	19	4434513	-	CHAF1A	+
JID.72	ART alone	19	7989092	-	CTXN1	-
JID.51	ART+IL2	19	10778431	-	ILF3	+
JID.55	ART+IL2	19	11014499	-	CARM1	+
JID.83	ART+IL2	19	11231786	+	LDLR	+
JID.74	ART alone	19	12245746	+	ZNF20	-
JID.25	ART+IL2	19	12394499	+	ZNF44	-
JID.83	ART alone	19	14210497	+	PRKACA	-
JID.15	ART+IL2	19	19595947	+	GATAD2A	+
JID.29	ART+IL2	19	30483403	-	C19orf2	+
JID.75	ART alone	19	36237881	-	PSENEN	+
JID.61	ART+IL2	19	42053987	-	CEACAM21	+
JID.47	ART+IL2	19	48679145	+	LIG1	-
JID.34	ART+IL2	19	49963399	-	ALDH16A1	+

Patient ID	Treatment	Chr	Int Locus	ISS strand	Ref Seq Symbl	Gene Ori
JID.47	ART+IL2	19	53351485	+	ZNF468	-
JID.72	ART alone	19	55619243	+	PPP1R12C	-
JID.79	ART alone	19	58024410	+	ZNF773	+
JID.19	ART+IL2	20	31442462	-	MAPRE1	+
JID.19	ART+IL2	20	36330726	+	CTNNBL1	+
JID.76	ART alone	20	56202504	+	ZBP1	-
JID.17	ART+IL2	20	58440603	+	SYCP2	-
JID.72	ART alone	20	62345446	-	ZGPAT	+
JID.50	ART+IL2	21	40172570	-	ETS2	+
JID.61	ART+IL2	21	45589844	+	C21orf33	+
JID.64	ART+IL2	22	20924426	-	MED15	+
JID.11	ART+IL2	22	42036572	-	XRCC6	+
JID.74	ART alone	22	45540666	-	NUP50	+
JID.73	ART alone	X	44380081	-	FUNDC1	-
JID.35	ART+IL2	X	129287692	+	AIFM1	-
JID.64	ART+IL2	X	153356673	+	MECP2	-

Patient ID: Patient Identifier
Treatment: type of treatment adopted: ART alone is used for patients that received a 3-drug combination based on a virus protease inhibitor plus 2 nucleoside reverse-transcriptase inhibitors, ART+IL2 indicates patients that received same ART regimen plus IL-2 administration as indicated in Tambussi et al, *The Journal of infectious diseases 2001*.
Chr: chromosome
Integration Locus: position on chromosome in nucleotides
ISS strand: orientation of the integrated virus with respect the chromosome: +, chromosome and vector are in the same orientation; -, vector sequence is in opposite orientation compared to the chromosome.
Ref Seq Gene Symbol: nearest gene targeted by viral insertion.
Gene Ori: orientation of the gene sequence with respect to chromosome

Supplementary Table 2. Comparison of the genomic distribution of our HIV-1 integration sites to the datasets of Ikeda et al, Maldarelli et al., Wagner et al., by Genomic Hyperbrowser analysis tool (v2.0b, 1 Mb intervals).

	Region	P-value	FDR-adjusted p-values	Number of IS in 'Our study'	Number of IS in 'Ikeda et al'
Comparison IKEDA et al vs Our study	chr6:90000001-100000000	5.89E-07	1.59E-05	4	43
	chr14:20000001-30000000	0.04521	0.4069	0	7
	chr22:10000001-20000000	0.04521	0.4069	0	7
Comparison Maldarelli et al vs Our study	chr9:1-10000000	0.02764	0.8811	3	4
Comparison Wagner et al vs Our study	-	-	-	-	-

Supplementary Table 3. Multiple targeted genes identified in different HIV-1 derived integration datasets

Maldarelli et al	N°ins	*Wagner* et al	N°ins	*Ikeda* et al	N°ins	Our study	N°ins
MKL2	23	BACH2	9	BACH2	43	STAT5B	4
BACH2	19	STAT5B	4	STAT5B	18	BACH2	3
STAT5B	10	MKL2	3	OR4E2	7	OPTC	2
TAOK1	7	SMG6	3	GNB1L	6	TBC1D5	2
DIP2A	7	ANKRD13C	2	SPATS2	4	SMARCC1	2
FCHSD2	6	C2CD3	2	EIF4G3	3	MAP4	2
TNRC6C	6	GCN1L1	2	GPBP1L1	2	UTRN	2
CYTH1	6	CREBBP	2	OXNAD1	2	C9orf102	2
RPTOR	6	KCTD13	2	FOXP1	2	ETV6	2
TNRC6B	6	IKZF3	2	CARD11	2	LDLR	2
PACS1	5	HNRNPUL1	2	NRF1	2		
KDM2A	5	TRAPPC10	2	PLEC1	2		
ETS1	5	MAPK1	2	UBAC2	2		
SMARCE1	5	APOBEC3C	2	TAOK1	2		
IER2	5	OXCT1	2	RPTOR	2		
GATAD2A	5	ST8SIA4	2	TYK2	2		
FKBP5	5	NSMCE2	2	TOP1	2		
UBE2H	5			AVPR2	2		
EIF4G3	4						
KIAA0319L	4						
VPS45	4						
SHOC2	4						
MLL	4						
CDKN1B	4						
PRKCB	4						
NFATC3	4						
NFAT5	4						
UBE2G1	4						
IKZF3	4						
STAT3	4						
FOXK2	4						
DNMT1	4						
CEACAM21	4						
MKL1	4						
ALG12	4						
MAP4	4						
PAK2	4						
NSD1	4						
OSBPL3	4						
FNBP1	4						

Supplementary Table 4. Comparison of the HIV-1/LV integration site profiles at BACH2 and STAT5B loci in different genomic integration studies.

BACH2	Integration study[A]	Sample Type[B]	Vector/virus[C]	Tot IS[D]	BACH2 Targeting frequency[E]	Insertion in same orientation[F]	P VS This study[G]	P VS IKEDA et al[H]	P VS WAGNER et al[I]	P VS MALDARELI et al[L]
HIV-derived vector integration datasets	Wang et al, *Mol Ther 2009*	T	SIN HIV-vector	7262	0.01% (N=1)	100% (N=1)	< 0.0001	< 0.0001	< 0.0001	< 0.0001
	Brady et al, *AIDS 2009*	T	X4-HIV-1 vector	2073	0.24% (N=5)	60% (N=3)	0.004	< 0.0001	< 0.0001	0.0009
	Cartier et al, *Science 2009*	HSPC	SIN HIV-vector	3520	0	-	< 0.0001	< 0.0001	< 0.0001	< 0.0001
	Biffi, Montini et al, *Science 2013*	HSPC	SIN HIV-vector	38475	0.02% (N=9)	67% (N=6)	< 0.0001	< 0.0001	< 0.0001	< 0.0001
	Aiuti et al, *Science 2013*	HSPC	SIN HIV-vector	33264	0.01% (N=2)	50% (N=1)	< 0.0001	< 0.0001	< 0.0001	< 0.0001
				Av.	0.057% ± 0.103					
HIV-1 virus integration datasets	This study	PBMC	HIV-1 virus	198	1.51% (N=3)	100% (N=3)	-			
	Ikeda et al, *JID 2007*	T	HIV-1 virus	294	14.63% (N=43)	100% (N=43)	< 0.0001	-		
	Wagner et al, *Science 2014*	PBMC	HIV-1 virus	387	2.32% (N=9)	100% (N=9)	NS	< 0.0001	-	
	Maldarelli et al, *Science 2014*	PBMC	HIV-1 virus	1723	1.10% (N=19)	100% (N=19)	NS	< 0.0001	NS	-
				Av	4.9% ± 6.5					

STAT5B	Integration study[A]	Sample Type[B]	Vector[C]	Tot IS[D]	STAT5B Targeting frequency[E]	Insertion in same orientation[F]	P VS This study[G]	P VS IKEDA et al[H]	P VS WAGNER et al[I]	P VS MALDARELI et al[L]
HIV-derived vector integration datasets	Wang et al, *Mol Ther 2009**	T	SIN HIV-vector	7262	0.12% (N=9)	33% (N=3)	< 0.0001	< 0.0001	< 0.0001	0.0002
	Brady et al, *AIDS 2009**	T	X4-HIV-1 vector	2073	0.43% (N=9)	22% (N=2)	0.0049	< 0.0001	NS	NS
	Cartier et al, *Science 2009**	HSPC	SIN HIV-vector	3520	0.11% (N=4)	50% (N=2)	< 0.0001	< 0.0001	0.0001	0.0002
	Biffi, Montini et al, *Science 2013**	HSPC	SIN HIV-vector	38475	0.18% (N=69)	49% (N=34)	< 0.0001	< 0.0001	0.0001	< 0.0002
	Aiuti et al, *Science 2013**	HSPC	SIN HIV-vector	33264	0.17% (N=58)	67% (N=39)	< 0.0001	< 0.0001	< 0.0001	0.0002
				Av.	0.21% ± 0.13					
HIV-1 virus integration dataset	This study*	PBMC	HIV-1 virus	198	2.01% (N=4)	75% (N=3)	-			
	Ikeda et al, *JID 2007**	T	HIV-1 virus	294	6.12% (N=18)	100% (N=18)	0.031	-		
	Wagner et al, *Science 2014*	PBMC	HIV-1 virus	387	1.03% (N=4)	25% (N=1)	NS	0.0003	-	
	Maldarelli et al, *Science 2014*	PBMC	HIV-1 virus	1723	0.58% (N=10)	50% (N=5)	0.025	< 0.0001	NS	-
				Av	2.44% ± 2.52					

A. The references of LV and HIV-1 integration studies are indicated. Asteriks indicate integration dataset in which the viral/genomic junction sequences have been reanalyzed using our bioinformatic pipeline (Calabria et al, 2014)
B. The cell type in which the integration study was performed is indicated: T cells (T), peripheral blood mononuclear cells (PBMC) and hematopoietic stem and progenitor cells (HSPC)
C. Type of virus or vector analyzed/used
D. Total number of unique integration sites (IS)
E. Targeting frequency. Percentatage of IS targeting *BACH2* or *STAT5B* with respect the total IS retrieved in each study, the number of vector/viral integrations targeting *BACH2* or *STAT5B* are reported in brackets. The average of the integration frequency (Av) was calculated for the virus or vector derived datasets
F. Insertion in same orientation. The relative percentage of the viral/vector integration targeting the BACH2 or STAT5B in the same orientation as gene transcription is reported percent. The number of IS with the same orientation are indicated
G. p values calculated by Chi square Analysis of Contingency comparing the STAT5B or BACH2 targeting frequency observed in our study versus all the indicated datasets.
H. p values calculated by Chi square Analysis of Contingency comparing the STAT5B or BACH2 targeting frequency observed in Ikeda's study versus all the indicated datasets
I. p values calculated by Chi square Analysis of Contingency comparing the STAT5B or BACH2 targeting frequency observed in Wagner's study versus all the indicated datasets
J. p values calculated by Chi square Analysis of Contingency comparing the STAT5B or BACH2 targeting frequency observed in Maldarelli's study versus all the indicated datasets

Supplementary Table 5. Patient demographics

Patient ID	Age (y)	Gender	HIV/STAT5B	HIV/BACH2	CDC classification	HIV/RNA copies/ml plasma	CD4+ (cells/ul)	CD4/CD8 ratio	Group	1st TP analyzed (y after ART)	2nd TP analyzed (y after ART)	3rd TP analyzed (y after ART)	HIV DNA (Log10 HIV genome/106 cells)	Current ART regimen
1	48	M			A3	36	220	0.30	NT	1.1	-	-	-	MVC+LPV/r
2	43	M			A2	36	565	0.58	NT	0.5	-	-	2.72	TDF/FTC+ATV/r
3	46	M			A2	36	558	0.63	NT	0.5	-	-	-	MVC+LPV/r
4	34	M			A1	36	521	0.47	NT	0.7	-	-	-	TDF/FTC+LPV/r
5	82	M	x		A2	36	462	0.56	NT	0.7	-	-	-	TDF/FTC+LPV/r
6	58	M	x		A2	36	696	1.14	NT	0.7	-	-	3.31	MVC+LPV/r
8	56	M	x		B2	36	566	0.50	NT	0.7	-	-	-	TDF/FTC+LPV/r
9	55	M			A2	36	616	0.68	NT	0.7	-	-	3.67	TDF/FTC+LPV/r
10	38	M			A2	36	727	0.77	NT	0.7	-	-	-	MVC+LPV/r
12	46	M	x		A2	36	448	0.72	NT	2.0	-	-	-	MVC+LPV/r
13	43	M			A2	36	536	0.66	NT	0.7	-	-	2.68	MVC+LPV/r
14	54	M			A3	36	491	0.62	NT	1.2	-	-	-	TDF/FTC+LPV/r
15	59	M			A2	36	401	0.38	NT	0.7	-	-	2.45	MVC+LPV/r
20	48	M			A2	36	594	0.29	NT	0.5	-	-	-	TDF/FTC+LPV/r
21	53	M	x		A2	36	522	0.66	NT	0.5	-	-	3.23	TDF/FTC+LPV/r
22	51	M			A3	36	682	0.40	NT	9.8	-	-	2.46	MVC+LPV/r+r
24	42	M			A2	36	288	0.25	NT	0.5	-	-	2.73	TDF/FTC+LPV/r
25	47	F	x		A3	36	668	0.27	NT	0.4	-	5	-	MVC+LPV/r
26	46	M			A2	62	540	0.49	NT	0.5	-	-	-	TDF/FTC+LPV/r
27	35	M			A2	36	592	0.71	NT	0.7	-	-	2.93	TDF/FTC+LPV/r
30	36	M			A2	36	308	0.43	NT	0.2	-	-	-	MVC+LPV/r
31	37	M			A2	364	488	0.31	NT	0.7	-	-	-	MVC+LPV/r
32	46	M	x	x	A2	36	707	1.64	NT	0.7	-	5	3.11	TDF/FTC+LPV/r

Patient ID	Age (y)	Gender	HIV/STAT5B	HIV/BACH2	CDC classification	HIV/RNA copies/ml plasma	CD4+ (cells/ul)	CD4/CD8 ratio	Group	1st TP analyzed (y after ART)	2nd TP analyzed (y after ART)	3rd TP analyzed (y after ART)	HIV DNA (Log10 HIV genome/106 cells)	Current ART regimen
33	36	M			A2	36	926	0.96	NT	0.7	-	-	3.82	MVC+LPV/r
34	40	M	x		A2	36	294	0.39	NT	1.4	-	-	-	TDF/FTC+LPV/r
36	40	M			A2	36	455	0.87	NT	0.4	-	-	2.68	TDF/FTC+LPV/r
37	26	M			A2	36	526	0.72	NT	1.4	-	-	3.05	TDF/FTC+LPV/r
38	33	M	x	x	A2	36	619	0.46	NT	0.5	-	4	-	TDF/FTC+LPV/r
41	31	M		x	A2	36	932	0.47	NT-PHI	1.6	-	-	2.85	TDF+FTC+EFV
42	52	M			A1	36	660	0.66	NT-PHI	1.4	-	-	-	ATV+RTV
43	43	M			A1	36	1619	1.02	NT-PHI	1.9	-	-	-	MVC+3TC+ddI
44	55	M			A1	36	800	1.27	NT-PHI	1.6	-	-	2.23	DRV+RTV
45	47	M			A1	36	876	1.22	NT-PHI	1.8	-	-	2.88	TDF/FTC+ATV+RTV
46	47	M			A1	36	861	2.00	NT-PHI	1.8	-	-	2.66	TDF/FTC+LPV/r
47	41	M	x	x	A2	36	1082	0.67	NT-PHI	1.2	-	-	3.04	TDF/FTC+LPV/r
48	39	M			A2	36	888	1.04	NT-PHI	1.9	-	-	2.77	DRV+RTV
49	36	M			A2	36	888	0.99	NT-PHI	1.5	-	-	2.30	TDF/FTC+ATV+RTV
50	34	F			A2	36	518	0.95	NT-PHI	1.6	-	-	3.10	MVC+RTV+DRV
51	38	M			A2	36	399	1.35	NT-PHI	1.8	-	-	3.47	DRV+RTV
52	43	M			A3	134	471	0.90	NT-PHI	1.9	-	-	-	MVC+LPV/r
53	44	M			A2	36	701	0.92	NT-PHI	1.6	-	-	3.04	TDF/FTC+ATV+RTV
54	57	M			A2	36	646	1.07	NT-PHI	1.4	-	-	4.24	TDF/FTC+ATV+RTV
55	34	M			A1	36	546	0.99	NT-PHI	1.9	-	-	3.39	TDF+FTC+EFV
56	45	M	x		A2	36	545	0.45	NT-PHI	2.0	-	3	2.66	TDF/FTC+DRV+RTV
57	44	M			A2	36	416	1.53	NT-PHI	2.7	-	-	2.95	RPV/TDF/FTC
58	44	M			A2	36	468	1.37	NT-PHI	2.7	-	-	2.42	3TC/ABC+RPV
59	39	M			A2	36	682	1.14	NT-PHI	2.0	-	-	-	DRV+RTV
63	48	M	x		A3	36	228	0.22	EXP	23.1	-	30	3.53	MVC+RAL

Patient ID	Age (y)	Gender	HIV/STAT5B	HIV/BACH2	CDC classification	HIV/RNA copies/ml plasma	CD4+ (cells/ul)	CD4/CD8 ratio	Group	1st TP analyzed (y after ART)	2nd TP analyzed (y after ART)	3rd TP analyzed (y after ART)	HIV DNA (Log10 HIV genome/106 cells)	Current ART regimen
64	75	M			C3	36	491	0.85	EXP	17.4	26.9	-	2.97	ETR+MVC+RAL
65	51	M	x		C3	36	755	0.75	EXP	13.2	21.4	-	2.94	ETR+MVC+RAL
66	58	M			C3	36	481	0.77	EXP	16.9	17.0	-	3.03	ETR+MVC+RAL+TDF/FTC
67	63	M			C3	36	502	0.30	EXP	14.0	20.7	-	2.46	ETR+MVC+RAL
68	53	M			C3	36	364	0.38	EXP	11.2	17.0	-	-	DRV+RTV+RAL+ETR+TDF/FTC
69	59	M	x		C3	36	203	0.20	EXP	13.5	15.0	-	2.51	ETR+RAL+DRV+RTV
70	55	M			C3	36	319	0.52	EXP	7.5	17.4	-	1.75	ETR+RAL+DRV+RTV
71	52	M	x	x	B3	4411	449	0.20	EXP	11.3	11.3	19	2.83	RPV/TDF/FTC
72	54	M	x		A2	244600	301	0.40	EXP	13.0	15.1	-	3.09	ETR+MVC+RAL
73	48	M			A1	36	1097	0.96	EXP	9.3	16.8	-	-	ETR+RAL+DRV+RTV
74	60	M	x		C3	36	485	0.45	EXP	13.0	13.0	-	2.69	ETR+RAL+DRV+RTV
75	53	F	x	x	B2	60	316	0.66	EXP	10.0	16.8	-	2.05	ETR+RAL+DRV+RTV
76	52	M	x		C3	36	419	0.66	EXP	7.5	13.8	15	2.99	MVC+RAL
77	49	M	x		C2	36	513	0.60	EXP	15.3	11.3	23	2.47	MVC+RAL
78	52	M		x	C3	36	619	1.20	EXP	14.0	19.2	-	2.97	MVC+RAL
79	52	M			C3	36	355	0.37	EXP	13.2	17.8	-	2.96	ETR+MVC+RAL
80	58	M	x		C2	36	561	0.70	EXP	15.9	16.7	-	2.66	ETR+MVC+RAL
81	48	M			A3	36	491	0.48	EXP	9.6	19.7	-	2.93	RAL+TDF/FTC
82	51	F			C3	36	691	0.58	EXP	13.6	13.6	-	2.28	ETR+MVC+RAL
83	50	M			C3	36	1088	0.99	EXP	15.3	17.4	-	3.72	MVC+RAL+TDF/FTC
84	69	M			C3	36	216	0.54	EXP	14.4	19.1	-	2.65	DRV+RTV+RAL+ETR+TDF/FTC
85	58	M			A3	36	829	0.44	EXP	12.0	18.0	-	2.56	ETR+MVC+RAL
86	69	M		x	A3	36	344	0.46	EXP	18.0	15.8	-	3.33	ETR+MVC+RAL
87	59	M			B3	36	587	0.40	EXP	11.3	20.8	-	2.93	ETR+RAL+DRV+RTV
88	49	M	x		A2	36	434	0.58	EXP	11.0	14.9	-	2.95	ETR+MVC+RAL

Patient ID	Age (y)	Gender	HIV/STAT5B	HIV/BACH2	CDC classification	HIV/RNA copies/ml plasma	CD4+ (cells/ul)	CD4/CD8 ratio	Group	1st TP analyzed (y after ART)	2nd TP analyzed (y after ART)	3rd TP analyzed (y after ART)	HIV DNA (Log10 HIV genome/106 cells)	Current ART regimen
89	50	M	x		C3	36	453	0.53	EXP	6.6	14.8	-	4.45	ETR+MVC+RAL
90	54	F			B3	36	700	0.72	EXP	17.1	10.4	-	2.83	ETR+MVC+RAL
91	54	F			A3	36	315	0.39	EXP	10.9	20.9	-	3.37	ETR+RAL+DRV+RTV
92	50	F	x		C3	36	67	0.76	EXP	16.9	14.9	-	-	ETR+RAL+DRV+RTV
93	55	M	x		C3	36	449	0.35	EXP	12.6	20.7	-	3.00	ETR+RAL+DRV+RTV
94	53	M			C3	47	465	0.35	EXP	16.1	16.4	-	4.41	ETR+MVC+RAL
95	50	M			B3	36	519	0.71	EXP	9.9	19.0	-	3.43	ETR+MVC+RAL
96	58	M	x		B2	36	784	0.47	EXP	16.6	13.3	25	2.63	ETR+MVC+RAL+TDF/FTC
97	72	M			B3	36	503	0.78	EXP	11.6	20.6	-	1.98	ETR+MVC+RAL
98	72	M			A3	48	384	0.32	EXP	10.4	15.4	-	2.75	ETR+MVC+RAL+TDF/FTC
99	55	M			A2	36	695	0.79	EXP	10.7	14.3	-	3.67	ETR+RAL+DRV+RTV
100	45	F			A3	39	324	0.64	EXP	12.2	14.1	-	1.42	ETR+RAL+DRV+RTV
101	56	M			C2	36	695	0.64	EXP	15.5	15.6	-	2.96	ETR+RAL+DRV+RTV
103_BA	-	M	x		-	36	-	-	NT	1.0	15.3	-	-	TDF/FTC+LPV/r

Patient ID: Patient Identifier

Age: Current Age of the patient (years)

Gender: Gender of the patient

HIV/STAT5B: the presence of the HIV/STAT5B chimeric transcript has been identified in the PBMC of the patient

HIV/BACH2: the presence of the HIV/BACH2 chimeric transcript has been identified in the PBMC of the patient

CDC classification: Categorization of HIV patients at the diagnosis, based on the U.S. Centers for Disease Control and Prevention (CDC) classification system which evaluate the severity of HIV disease by CD4 cell counts and by the presence of specific HIV-related conditions.

HIV RNA copies: HIV RNA copies/ml measured in the plasma of the HIV patient prior at the first time point analyzed

CD4 (cells/ul): CD4 cells count /ul at the first time point analyzed

CD4/CD8 ratio: Ratio of CD4+ and CD8+ cells found in the blood of HIV patients at the first time point analyzed

Group: Patients divided in subgroups depending on the history and type of ART regimen. Experience (EXP) indicates patients that underwent both Nucleoside and Non-nucleoside reverse transcriptase inhibitors (NRTIs and NNRTIs, respectively); New Therapy (NT) indicates patients that experienced ART regimen based only on NNRTIs. NT-PHI indicates patients treated with NNRTIs ART regimen and that are in a Primary phase of HIV infection (PHI).

1st time point analyzed (y after ART): 1 st Molecular Analyses performed on PBMC isolated from the HIV patient post ART regimen

2nd time point analyzed (y after ART): 2nd Molecular Analyses performed on PBMC isolated from the HIV patient post ART regimen

3rd time point analyzed (y after ART): Molecular Analyses performed on sorted cells isolated from selected HIV patient post ART regimen

HIV DNA: copies of the HIV DNA evaluated on total PBMC from the 1st or 2nd time point analyzed and measure as Log10 of the HIV genome on 106 cells analyzed

Current ART regimen: current ART regimen adopted.

In blue are highlighted patients from whom we purified and sorted different hematopoietic cell subsets.

Supplementary Table 6. Haplotypes identified within the amplified chimeric HIV/STAT5B bands

Sample[(a)]	Cell Type[(b)]	Tot Reads[(c)]	Num. Clones[(d)]	Rel. Freq. Clones[(e)]
ID_77	Tcm	2002	1	1
	Teff	2711	1	1
	Treg	1214	2	0.39
				0.61
	PBMC	620	1	1
ID_96	Tcm	34945	2	0.52
				0.48
	Treg	5788	1	1
	PBMC	15836	1	1
ID_25	Teff	22441	2	0.59
				0.41
	Treg	37010	1	1
ID_56	Treg	1799	1	1
ID_76	Treg	5334	1	1
ID_63	Treg	2242	1	1
ID_65	PBMC	825	2	0.29
				0.71
ID_75	PBMC	755	2	0.32
				0.68
ID_12	PBMC	4821	2	0.4
				0.6
ID_21	PBMC	658	1	1
ID_32	PBMC	1948	1	1
ID_34	PBMC	2429	1	1
ID_38	PBMC	11148	1	1
ID_72	PBMC	2027	1	1
ID_74	PBMC	5620	1	1
ID_80	PBMC	698	1	1

(a) Sample: Sample ID number
(b) Cell Type: Cell type from which the chimeric band was amplified
(c) Tot reads: total number of reads that were aligned for that sample
(d) Num Clones: number of different haplotypes identified within each sample
(e) Freq. Clones: Relative abundance of each clone identified within the analysed sample

SUPPLEMENTARY METHODS

Cloning procedures for LV Transfer plasmids

The bidirectional SIN.LV.PGK.BACH2 and SIN.LV.PGK.STAT5B transfer plasmid were produced from the SIN.LV.PGK.GFP.PRE[1] using Gateway Technology. Briefly, SIN.LV.PGK.GFP.PRE plasmid was cut with BamHI/SalI to excise the GFP coding sequence, blunt-filled, dephosphorylated and ligated to a blunt-ended cassette containing the att_RfA sites of the Gateway system (Gateway®Vector Conversion System with One Shot ®ccdBSurvival™ 2T1R Competent Cells, Invitrogen) to generate a SIN.LV. PGK.Gateway_RfA plasmid. Next, the resulting SIN.LV. PGK.Gateway_RfA plasmid was linearized with XhoI and ligated with an XhoI fragment containing the mhCMV.mOrange.pA sequence obtained from the pCCLsin.cPPT.SFFV.tTR-KRAB.WPRE.mhCMV.mOrange.pA plasmid (kindly provided by Angelo Lombardo). Finally, we used specific ORFEXPRESS™ Gateway® Shuttle Clone (Labomics) containing sequencing validated cDNA for *BACH2* and *STAT5B* gene to perform the L+R Gateway reaction with our receiving bidirectional SIN.LV Gateway_RfA. These transfer plasmids were subsequently used for vector production. SIN.LV.PGK.GFP/dNGFR, vector was previously described [2,3]

Gene expression analysis

Nested PCR was performed in a standard thermal cycler machine (Bio-Rad) and PCR conditions are the following: 1st PCR cycle, 95 °C for 2’, 40 cycles of 95 °C for 30’’, 58 °C for 60’’, 72°C for 90’’, and 72 °C for 10’; 2nd PCR cycle, 95 °C for 2’, 40 cycles of 95 °C for 30’’, 58 °C for 60’’, 72°C for 30’, and 72 °C for 10’.

The oligoes used for the nested PCR are the following:

STAT5B I 5' CATTGTTGGCTTCTCGGACC 3'

STAT5B II 5' GGGCAGCGGTCATACGTG 3'

BACH2 I 5' CAGCTTGGCAGTGTAGGC 3'

BACH2 II 5' CCCTGGCTGTGACCTCCTC 3'

HIV-I 5' GAGCTGTCTGGCTAACTAGG 3'

HIV-II 5’ AGCTTGCCTTGAGTGCTTCA 3’

droplet digital PCR (ddPCR) analyses

Specific and custum-made ddPCR assays were designed for the detection of the chimeric HIV/*STAT5B* and HIV/*BACH2* transcripts. Ten nanograms of cDNA were used for PCR amplification performed in duplicate and in a final volume of 20 ul. Approximately up to 20,000 monodispersed droplets for each sample were prepared using the QuantaLife droplet generator. The droplets were transferred to a 96-well PCR plate and amplified to endpoint in a standard thermal cycler (Bio-Rad) using the following conditions: for HIV/*STAT5B* and HIV/*BACH2* chimeric transcripts: 95 °C for 10’, 40 cycles of 94 °C for 30’’, 65 °C for 60’’, and 98 °C for 10’; for HPRT and STAT5B transcript: 95 °C for 10 min, 40 cycles of 94 °C for 30’, 60 °C for 60’’, and 98 °C for 10’.

The custom-made dd-PCR assays designed for the detection of the chimeric HIV/*STAT5B* and HIV/*BACH2* transcripts have the following sequence:

HIV/*BACH2*

Forward primer: TAGCAGTGGCGCCCGAA

Reverse primer: CTTCTCCTTGGAGCTGCTG

Probe: ATTGTAAACCATGGCTGTGTGGATACAAG

HIV/*STAT5B*

Forward primer: TCTCTAGCAGTGGCGCCC

Reverse primer: GCTTCTCATCCACAGACAT

Probe: TCAGCAAGCCGAGTCCTGCGTCGAGA

On selected samples, Illumina barcodes were attached to the amplified products using TruSeq Nano DNA LT Sample Prep Kit (Illumina), and the different libraries were pooled and sequenced using an Illumina

MySeq platform. Next-Generation Sequencing reads were then aligned against a reference sequence obtained by joining the HIV sequence spanning from the U5 to the major SD signal with the second exon of the human STAT5B gene. The alignment against this gene-vector junction reference sequence has been performed using BWA[4] with standard parameters for paired-end sequences (Suppl. Table 6). The resulting alignments were filtered using the Picard software (http://broadinstitute.github.io/picard) and the MarkDuplicate function that allow the depletion of optical duplicates (reads originated from a single DNA fragment). The different HIV haplotypes were then identified among the different reads using the PredictHaplo software[5]. Briefly, this procedure has been designed to infer the number and frequency of the different haplotypes in a sample containing a mixture of HIV clones. PredictHaplo takes advantage of a Dirichlet Process Mixture Model to identify sequence variations. Clustal Omega[6] with standard parameters was then used to create a multiple sequence alignment of clones/haplotypes reference sequences. Finally, phylogenetic tree were generated using a Neighbour-joining based procedure with ClustalW2[7] and represented with the FigTree software (http://tree.bio.ed.ac.uk/).

SUPPLEMENTARY REFERENCES

1 Follenzi, A., Ailles, L. E., Bakovic, S., Geuna, M. & Naldini, L. Gene transfer by lentiviral vectors is limited by nuclear translocation and rescued by HIV-1 pol sequences. *Nat Genet* **25**, 217-222, doi:10.1038/76095 (2000).

2 Amendola, M., Venneri, M. A., Biffi, A., Vigna, E. & Naldini, L. Coordinate dual-gene transgenesis by lentiviral vectors carrying synthetic bidirectional promoters. *Nat Biotechnol* **23**, 108-116, doi:nbt1049 [pii]10.1038/nbt1049 (2005).

3 Montini, E. *et al.* The genotoxic potential of retroviral vectors is strongly modulated by vector design and integration site selection in a mouse model of HSC gene therapy. *J Clin Invest* **119**, 964-975, doi:10.1172/JCI37630 (2009).

4 Li, H. & Durbin, R. Fast and accurate short read alignment with Burrows-Wheeler transform. *Bioinformatics* **25**, 1754-1760, doi:10.1093/bioinformatics/btp324btp324 [pii] (2009).

5 Prabhakaran, S., Rey, M., Zagordi, O., Beerenwinkel, N. & Roth, V. HIV Haplotype Inference Using a Propagating Dirichlet Process Mixture Model. *IEEE/ACM Trans Comput Biol Bioinform* **11**, 182-191, doi:10.1109/TCBB.2013.145 (2014).

6 Sievers, F. *et al.* Fast, scalable generation of high-quality protein multiple sequence alignments using Clustal Omega. *Mol Syst Biol* **7**, 539, doi:10.1038/msb.2011.75msb201175 [pii] (2011).

7 Larkin, M. A. *et al.* Clustal W and Clustal X version 2.0. *Bioinformatics* **23**, 2947-2948, doi:btm404 [pii]10.1093/bioinformatics/btm404 (2007).
